# Merging transcriptomics and metabolomics - advances in breast cancer profiling

**DOI:** 10.1186/1471-2407-10-628

**Published:** 2010-11-16

**Authors:** Eldrid Borgan, Beathe Sitter, Ole Christian Lingjærde, Hilde Johnsen, Steinar Lundgren, Tone F Bathen, Therese Sørlie, Anne-Lise Børresen-Dale, Ingrid S Gribbestad

**Affiliations:** 1 Department of Genetics, Institute for Cancer Research, Division of Surgery and Cancer, Oslo University Hospital Radiumhospitalet, Oslo, Norway; 2 Department of Circulation and Medical Imaging, Norwegian University of Science and Technology (NTNU), Trondheim, Norway; 3 Biomedical Research Group, Department of Informatics, University of Oslo, Oslo, Norway; 4 Department of Oncology, St. Olavs University Hospital Trondheim, Trondheim, Norway; 5 Institute of Clinical Medicine, Faculty of Medicine, University of Oslo, Norway

## Abstract

**Background:**

Combining gene expression microarrays and high resolution magic angle spinning magnetic resonance spectroscopy (HR MAS MRS) of the same tissue samples enables comparison of the transcriptional and metabolic profiles of breast cancer. The aim of this study was to explore the potential of combining these two different types of information.

**Methods:**

Breast cancer tissue from 46 patients was analyzed by HR MAS MRS followed by gene expression microarrays. Two strategies were used to combine the gene expression and metabolic data; first using multivariate analyses to identify different groups based on gene expression and metabolic data; second correlating levels of specific metabolites to transcripts to suggest new hypotheses of connections between metabolite levels and the underlying biological processes. A parallel study was designed to address experimental issues of combining microarrays and HR MAS MRS.

**Results:**

In the first strategy, using the microarray data and previously reported molecular classification methods, the majority of samples were classified as luminal A. Three subgroups of luminal A tumors were identified based on hierarchical clustering of the HR MAS MR spectra. The samples in one of the subgroups, designated A2, showed significantly lower glucose and higher alanine levels than the other luminal A samples, suggesting a higher glycolytic activity in these tumors. This group was also enriched for genes annotated with Gene Ontology (GO) terms related to cell cycle and DNA repair. In the second strategy, the correlations between concentrations of *myo*-inositol, glycine, taurine, glycerophosphocholine, phosphocholine, choline and creatine and all transcripts in the filtered microarray data were investigated. GO-terms related to the extracellular matrix were enriched among the genes that correlated the most to *myo*-inositol and taurine, while cell cycle related GO-terms were enriched for the genes that correlated the most to choline. Additionally, a subset of transcripts was identified to have slightly altered expression after HR MAS MRS and was therefore removed from all other analyses.

**Conclusions:**

Combining transcriptional and metabolic data from the same breast carcinoma sample is feasible and may contribute to a more refined subclassification of breast cancers as well as reveal relations between metabolic and transcriptional levels.

See Commentary: http://www.biomedcentral.com/1741-7015/8/73

## Background

Transcriptomics and metabolomics in cancer research have traditionally been considered as two separate fields. Different levels of the molecular processes are studied, aiming at improving cancer treatment by understanding the underlying mechanisms of the disease. Breast cancer treatment decisions today are mainly based on tumor size, histological characterization, grading and receptor status, as well as axillary lymph node status, and age of the patients [1]. However, patients with similar diagnosis and treatment can experience large differences in the progression and relapse of their disease. The various -omics fields, transcriptomics in particular, have provided an understanding of breast cancer as a group of molecularly distinct neoplastic disorders [2]. Clinical use of molecular characterization of breast cancer has the potential to stratify breast cancer patients for more individual treatment, but has so far only been implemented to a limited extent.

The field of transcriptomics, using DNA microarrays that enable measurements of thousands of RNA transcripts in a single experiment, has had a huge impact on breast cancer research over the last decade [2]. One of the important findings has been the classification of breast cancer into five subtypes (luminal A, luminal B, basal-like, ERBB2 enriched and normal-like) based on gene expression profiles of so called intrinsic genes [3,4]. This molecular subtyping of breast cancer, has been reproduced in several studies and is also associated with clinical outcome across datasets [5,6].

Metabolomics studies the metabolites and how they are affected by specific cellular processes. The possibility of using *in vivo *magnetic resonance spectroscopy (MRS) as a diagnostic, prognostic or predictive tool in the clinic simultaneously with an MR imaging (MRI) examination, makes MRS techniques attractive methods for molecular classification of disease. Metabolic profiling of intact biological samples using high resolution magic angle spinning magnetic resonance spectroscopy (HR MAS MRS) enables measurement of multiple cellular metabolites simultaneously. The method has been utilized in a wide range of biological applications [7], and studies of cancers have proven HR MAS MRS to be a promising tool in cancer diagnosis and treatment monitoring [8]. Importantly, the sample is kept intact throughout the HR MAS MRS analysis and can subsequently be analyzed by gene expression analysis.

Profiling gene expression and metabolite content in the same breast carcinoma samples enables comparisons of molecular findings at different levels. Gene expression data and metabolite data from MRS techniques of different samples from the same breast cancer cell line or xenograft model have been combined previously [9-12], but these studies have mainly focused on specific genes involved in choline metabolism, known as the Kennedy pathway. Combining transcriptomic and metabolomic profiling of the same sample allow us to capture a comprehensive picture at a given moment in time. Such studies could reveal differences and similarities between groups of samples at different molecular levels and provide a fundament for enhanced knowledge of the biological dynamics of breast cancers.

The aim of this study was to combine gene expression microarrays and HR MAS MRS for more refined profiling of breast tumors, and to explore some of the potentials and limitations of the experimental procedures. This study focuses on the most prevalent type of breast cancer, invasive ductal carcinomas (IDC) with oestrogen (ER) receptor positive disease, and the largest molecular subgroup within these tumors, luminal A. ER positive breast cancer accounts for approximately 2/3 of the cases, and although they overall have a relatively good prognosis, some patients experience relapse and do not respond to antieostrogen treatment. So far there are no biomarkers available to identify those patients and no targets for therapy, and identification of molecular markers of such tumors, possibly by metabolic profiling, are therefore of high relevance. Identifying subgroups of patients within this group is an important goal to make it possible to further individualize cancer diagnosis and treatment.

## Methods

### Patients

Tissue samples were selected from a local breast cancer tissue bank, obtained from patients with palpable breast lesions who underwent scheduled surgery for breast cancer at St. Olavs University Hospital in Trondheim, Norway (March 2000 - March 2004). Samples were placed in cryogenic vials and immersed in liquid nitrogen immediately after dissection. In this study we selected samples from patients diagnosed with IDC, who did not receive any treatment prior to surgery. Clinical data of the patients, such as diagnosis, tumor grade, tumor size, hormone receptor status and lymph node involvement were obtained from patient records, including pathology reports, and are summarized in Table 1. The Regional Committee for Medical and Health Research Ethics approved the study protocol, and all patients provided written informed consent.

**Table 1:** Clinical data on the 46 patients included in the main study.

Characteristics	Summary
Age, mean (range) years	64 (30 - 91)
Tumor size, mean (sd) (cm)	2.5 (± 1.4)
Grade	
I	5
II	17
III	21
NA	3
Axillary lymph node status	
Negative	22
Positive	20
NA	4
Oestrogen receptor (ER) status	
Negative	5
Positive	41
Progesterone receptor (PgR) status	
Negative	9
Positive	36
NA	1

All patients were diagnosed with invasive ductal carcinoma (IDC). All characteristics, except for age and tumor size, are summarized by number of patients in each group. ER and PgR were determined by immunohistochemistry, and samples with ≥10% staining cancer cells were considered receptor positive.

### Sample preparation and experimental design

**Figure 1:**
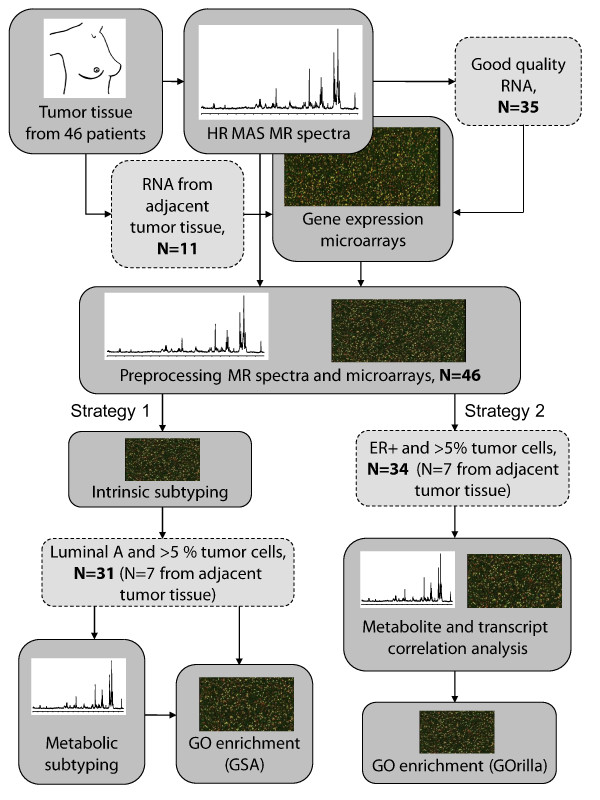
**An illustration of the experimental and data analysis workflow**. Experimental design and the two strategies of combining microarray and HR MAS MRS data are illustrated schematically.

An illustration of the experimental workflow as well as the data analysis for this study is shown in Figure 1. Tumor tissue samples from 46 patients were cut from each tumor and analyzed by HR MAS MRS. Proceeding HR MAS MRS, the tissue was directly snap frozen and later used to extract RNA for microarray analysis. For 11 of the tissue samples, the quality of RNA obtained after HR MAS MRS analysis was of insufficient quality, and adjacent tissue from the same tumors, not subjected to HR MAS MRS, was used for RNA extraction and microarray analysis. Additionally, histopathology was performed on adjacent tumor tissue for each sample, as described previously [13], to assess the percentage of cancer cells in each sample. The samples contained on average 23% tumor cells with a range of 0-80%.

### HR MAS MRS experiments

Sample preparation and HR MAS MRS experiments were performed as previously described [13]. In brief, all samples were cut to fit a 4 mm o.d. rotor with inserts (total sample volume 50 μL). Samples analyzed by HR MAS MRS weighed 18.6 mg in average (7.7 to 27.1 mg). The remaining rotor volume was filled with buffer (phosphate buffered saline (PBS) in D_2_O containing trimethylsilyl tetradeuteropropionic acid (TSP)). HR MAS MRS experiments were performed on a Bruker Avance DRX600 spectrometer equipped with a ^1^H/^13^C MAS probe (Bruker BioSpin GmbH, Germany). Samples were spun at 5 kHz while keeping the temperature at 4°C. Two types of one-dimensional ^1^H spectra were recorded, a single pulse experiment with water suppression and a spin-echo experiment (CPMG) for suppression of broad signals from macromolecules. Spectral assignments were performed based on previous HR MAS MRS studies of breast cancer lesions [13,14].

### RNA extraction and microarrays

Total RNA was extracted from the biopsies that were used in the HR MAS MRS procedure from each patient, following the protocol for the RNeasy Mini Kit (Qiagen, USA). High quality RNA, measured by BioAnalyzer 2100 (Agilent Technologies, USA) was used in microarray experiments, using 44k two-color Agilent Human Whole Genome Oligo Microarrays. The microarray analysis was performed according to the manufacturer's protocol, using Cy5 labeling for the tumor RNA and Cy3 for the reference RNA, Universal Human Reference (Stratagene, USA). Data were extracted from the microarray images using Feature Extraction Software v8.5 (Agilent Technologies, USA). The final dataset included gene expression microarray data from 46 patients, 35 obtained from RNA extracted from the HR MAS MRS analyzed tissue and 11 from adjacent tumor tissue, as illustrated in Figure 1.

### Preprocessing and normalizing the microarray data

The two-color microarray data were preprocessed and analyzed in R (v2.8) and Bioconductor, using the Feature Extraction text files. The red (sample) and green (reference) signals were set to be g(r)MeanSignal, and the data were normalized using the Limma package [15]. Each microarray was background corrected by subtracting the g(r)SpatialDetrendSurfaceValue values, and normalized with loess normalization. Normalization between arrays was performed with Gquantile normalization, which ensures the same empirical distribution for the green channel and adjusts the red channel accordingly. The data were log2 transformed and missing data were imputed using local least squares imputation from the pcaMethods package [16]. Control probes and probes which were flagged as outliers on more than 20% of the arrays were removed. The average of duplicate probes was calculated, and the averaged probe value with the highest IQR was picked to represent each transcript, when the gene symbol (supplied by Agilent) was represented by different probes. A subset of 1199 probes that were identified to have an altered gene expression after HR MAS MRS in a parallel study described below were also removed.

### Subtyping with microarray and HR MAS MRS data

Using gene median centered microarray data, the expression of "intrinsic" genes of the tumors from all 46 patients were analyzed for Spearman's correlation to published centroids of the five intrinsic subtypes [17]. The samples were classified according to the highest correlation value to the centroids. Prior to subtyping, probes of bad quality and 9 transcripts identified to show altered gene expression after HR MAS MRS were removed from the centroids, resulting in a match of 286 out of 306 intrinsic genes. The threshold for classification into a subtype was set to be correlation above 0.1 (One sample showed the same correlation to two subtypes and was regarded as unclassified). The high dimensional spin echo spectra (spectral region 4.8 - 1.45 ppm, excluding lipid containing regions at 2.8 ppm and 2.05 ppm, described by 5993 data points) of the 31 tumors classified as luminal A and with tumor cell percentage above 5%, were used to assess if adding the metabolic data revealed additional structure within this group of tumors. Normalization of the selected spectral regions was performed by mean normalization. Unsupervised hierarchical clustering with complete linkage was performed using Spearman's correlation as similarity measure, identifying three luminal A groups by cutting the dendrogram at height 0.39, which was decided as a threshold that gave convincing separation between groups. Multidimensional scaling (MDS) using Spearman's correlation as similarity measure was also used to assess if the same samples grouped together when using a different multivariate analysis. To test for metabolic differences between the three groups, t-tests were performed between pairs of points in the spectra. Differential gene expression analysis between the three identified luminal A groups was performed using the Limma package (R/Bioconductor) [15]. Modified t-tests were performed on each gene, with Empirical Bayes correction of the test statistics and Benjamini and Hochberg adjusted p-values. To assess if sets of genes known to be involved in the same process, share the same function or location were concordantly differentially expressed between the three luminal A groups, Gene Ontology (GO) [18] enrichment analyses were performed using Gene Set Analysis (GSA) [19]. GSA is a refined version of the original Gene Set Enrichment Analysis, which can be used to test if sets of genes are differentially expressed between two groups, using a maxmean statistic and permutation analysis to calculate the p-values. The GSA analysis was run with 10000 permutations, and the raw p-values were used. The Benjamini Hochberg false discovery rate was calculated separately.

### Correlation analysis between metabolite concentration and gene expression

Microarray and HR MAS MRS data from the same samples of the ER+ tumors with tumor cell percentage > 5% (N = 34) were used to test for nonzero correlations between metabolite and gene expression levels (For seven of the samples, the microarray data were obtained from adjacent tumor tissue). Quantification of eight tissue metabolites was performed based on peak areas in the single-pulse spectra and TSP concentrations as in [13]. After preprocessing the HR MAS MR spectra (WINNMR, Bruker Biospin GmbH, Germany), peak areas were calculated for the signals arising from *myo*-inositol, taurine, choline, GPC, PCho, glucose, creatine and glycine using the Voigt (combined Lorentzian and Gaussian) area function in PeakFit (Seasolve, MA USA). Quantification of lactate was not performed since lipid signals concealed the lactate signals (signals from lipid -CH_2 _overlap with lactate CH_3 _and glycerol backbone of triglycerides overlap with lactate CH) in two thirds of the spectra. Probes on the microarray with IQR < 0.8 were removed, and Spearman correlation tests between the 8 metabolites and all of the 13875 unique probes were performed. For each metabolite, all transcripts were ranked according to Spearman's rank correlation coefficient. The resulting genelists were analyzed for enriched GO-terms using GOrilla, which searches for GO-terms that are enriched in the top of the list compared to the rest of the list using the mHG statistics [20,21].

### Assessing the effect of HR MAS MRS on RNA integrity and transcription

No apparent differences were observed between the microarray data from the 35 samples that did undergo HR MAS MRS compared to the 11 samples that did not. However, to explore this issue more thoroughly, an additional study was designed to assess the feasibility of performing gene expression microarrays and HR MAS MRS on the same sample as well as identify transcripts that should be filtered out from microarray analyses if they have been systematically affected by the HR MAS MRS procedure. This additional study included 18 pairs of tumor samples, obtained from the local breast cancer tissue bank described above. One sample from each pair was analyzed by HR MAS MRS, RNA was isolated from both samples from each of the 18 pairs, and gene expression microarray analysis was performed. The HR MAS MRS experiments were performed as described above, while the RNA extraction, microarray experiments and preprocessing of the microarray data were performed using slightly different protocols, as described in Additional file 1: Supplementary documentation of exploring the effect of HR MAS MRS on RNA integrity and transcription. The effect of HR MAS MRS on RNA integrity was tested using a paired t-test on the RIN-values measured using Bioanalyzer 2100 (Agilent Technologies). Unsupervised hierarchical clustering of the normalized microarray data from the 18 pairs of samples and modified t-tests were performed [15] to test for differential gene expression caused by the HR MAS MRS procedure. The transcripts that showed significantly higher or lower (fdr < 0.01) expression in the samples analyzed by HR MAS MRS, were tested for enriched GO-terms [22]. Transcripts identified as having significantly altered expression after HR MAS MRS were removed from the microarray data in the main study. A more detailed description of the data analysis from this additional study can be found in Additional file 1: Supplementary documentation of exploring the effect of HR MAS MRS on RNA integrity and transcription.

## Results

Two main strategies of combining information from the microarray data with the HR MAS MR spectra are presented, as shown in the workflow illustrated in Figure 1. The first strategy uses multidimensional gene expression microarray data and the HR MAS MR spectra to identify molecular subgroups of tumors. The second strategy compares the quantified concentration of specific metabolites to the expression level of each transcript in the filtered microarray data. In a parallel study, we performed additional experiments to assess the impact of sample treatment during HR MAS MRS on RNA integrity and gene expression.

### Transcriptional and metabolic subtyping based on high-dimensional data

**Figure 2:**
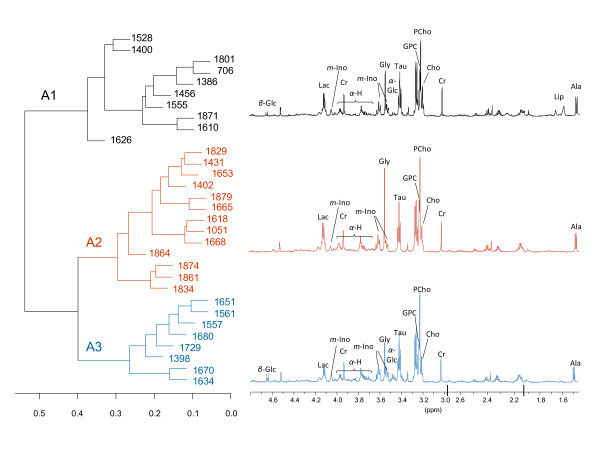
**Metabolic subtyping of luminal A samples using hierarchical clustering**. The HR MAS MR spectra of the samples classified as luminal A (N = 31), cluster in three groups using hierarchical clustering with Spearman's correlation as the distance measure and complete linkage. The mean spectra of each luminal A group are shown. Selected metabolite allocations are indicated with abbreviations (*β*-Glc: *β*-glucose; Lac: lactate; Cr: creatine; *m-*Ino: *myo*-inositol; *α*-H: *α*-H of amino acids; Gly: glycine; *α*-Glc: *α*-glucose; Tau: taurine; Cho: choline; GPC: glycerophosphocholine; PCho: phosphocholine; Ala: alanine). The spectral chemical shift-scale is non-continuous due to deletion of signals from lipid residues at 2.8 ppm and 2.05 ppm.

**Figure 3:**
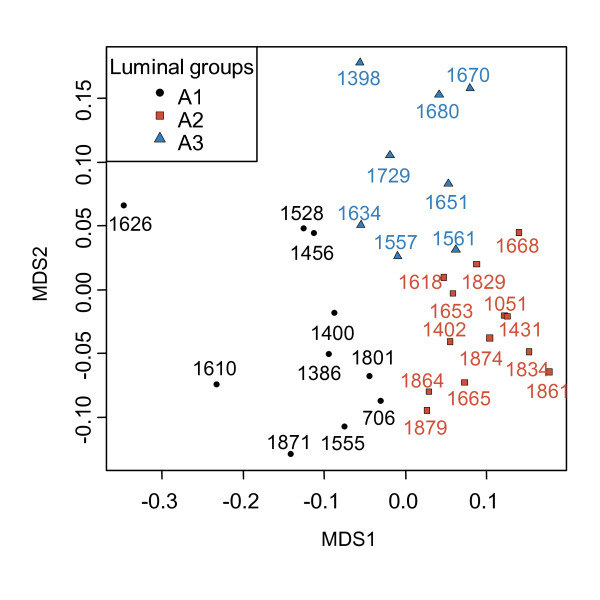
**Multidimensional scaling of the HR MAS MR spectra of the luminal A samples**. Multidimensional scaling of the HR MAS MR spectra of 31 luminal A samples, colored according to the A1-A3 subgroups found by hierarchical clustering.

**Figure 4:**
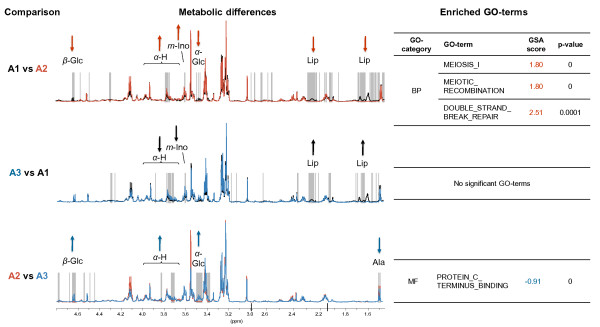
**Differences in metabolic and gene expression profiles of identified groups of luminal A samples**. Mean spectra of the A1 (black), A2 (red) and A3 (blue) groups of luminal A tumors are plotted with the significantly different points (p < 0.001) indicated with grey vertical lines. At ppm values with allocated metabolites and significant differences in one group versus the other, the direction of the arrows indicate if the latter group has the highest (up) or lowest (down) mean value. GO-terms that were significantly enriched (fdr < 0.01) in the different groups are indicated for each comparison, calculated using Gene Set Analysis (GSA) on the microarray data.

Each of the 46 tumor samples (Figure 1), were subtyped according to the highest Spearman's correlation of the expression of intrinsic genes to published centroids from the five subtypes [17]. This subtyping method resulted in the tumors being classified into 36 luminal A, 7 normal-like, 1 ERBB2 enriched, 1 basal-like, and 1 unclassified. The samples classified as luminal A were all ER positive. The ER-negative samples were classified as 1 basal-like and 4 normal-like. The HR MAS MR spectra from the tumors classified as luminal A (n = 31 with tumor cell percentage >5%), were used to search for diversity within this subgroup in the metabolic data. Hierarchical clustering based on the HR MAS MR spin echo spectra revealed three clusters that were convincingly separated when selecting a threshold of the dendrogram at height 0.39 (Figure 2). The samples belonging to each of these three subgroups of luminal A samples, designated A1, A2 and A3, also clustered together when applying MDS to the HR MAS MR spectra (Figure 3). Differences in the metabolic and gene expression profiles between the A1-A3 groups of luminal A tumors are illustrated in Figure 4. The metabolites allocated at the points that significantly differed between the profiles (p < 0.001) include α-glucose, β-glucose, amino acids (signals from α-H which is the Hydrogen bonded to the α-C of all amino acids), *myo*-inositol, alanine and lipid residues [14]. Glucose signals were significantly lower in the A2 group compared to the A1 and A3 luminal tumors. The α-H amino acid signals were significantly lower in A1 and higher in A3, compared to A2. Alanine signals were significantly higher in A2 compared to A3. Signals from lipid residues were significantly higher and signals from *myo*-inositol were significantly lower in A1 than in A2 and A3. Differential gene expression analysis between the three luminal A groups, using Limma, resulted in no significant differences in expression of single genes with fdr < 0.01. However, when analyzing differences in gene expression between sets of genes annotated with the same GO-terms, using GSA, different significantly enriched GO-terms (fdr < 0.01) were revealed by comparing the three luminal A groups (Figure 4). The A2 tumors were enriched for biological processes related to the cell cycle and DNA repair, namely "Meiosis I", "Meiotic recombination" and "Double strand break repair" compared to the A1 tumors. The A3 group was enriched for the molecular function "Protein C terminus binding" compared to the A2 group. There was not significant evidence of differences in tumor percentage between the three groups.

### Correlating metabolic and transcriptional profiles

**Figure 5:**
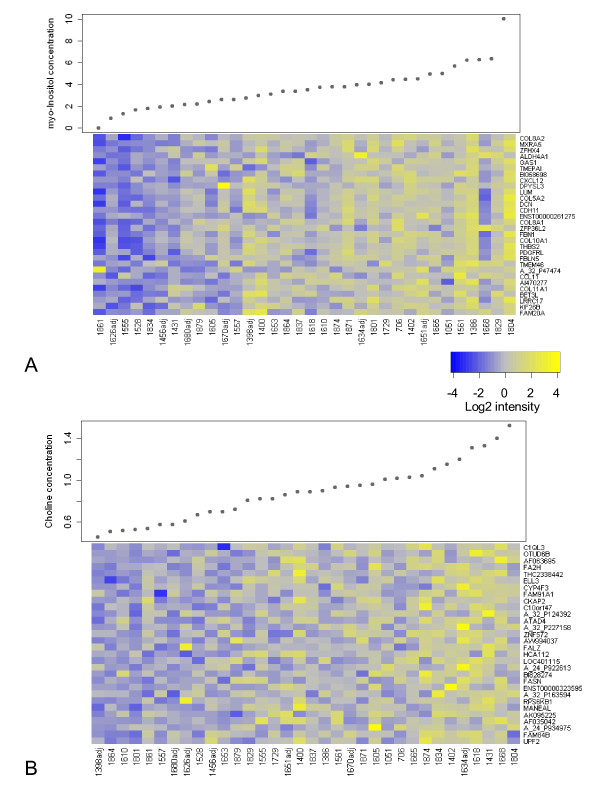
**Correlation analysis for *myo*-inositol and choline**. The concentration of (A) *myo*-inositol and (B) choline is plotted for the ER positive samples (n = 34), which are ordered according to concentration. The expression of the 30 transcripts that correlated most to these metabolites are shown as heatmaps (see color bar for scale), ordered according to Spearman's rank correlation coefficient. The samples for which the microarray data were obtained from RNA extracted from adjacent tumor tissue are labeled with the prefix "adj".

The levels of 8 specific metabolites were compared with the transcriptional activity in each tumor in the ER+ samples (n = 34 with tumor cell percentage >5%). These samples include the 31 luminal A samples mentioned above plus three samples that were classified as normal-like, but also correlated to the luminal A centroid. Tissue concentrations of some of the 8 quantified metabolites co-varied, see Additional file 2: Scatterplot of metabolite concentrations and tumor percentage. Taurine, *myo*-inositol and choline correlated significantly with each other, as did creatine, PCho, GPC and glycine. Glucose showed a negative or no significant correlation to all the other metabolites. Lists of all transcripts on the microarray with IQR > 0.8 (13875 unique probes) were ranked according to correlation, from high to low, to each of the 8 metabolites. Plots showing the co-variation of *myo-*inositol and choline concentration with the expression of the transcripts that correlated the most to these metabolites are shown in Figure 5. The ranked lists of transcripts were used to test for GO enrichment towards the top of the list, using GOrilla [20,21]. Significantly enriched (fdr < 0.003) GO-terms in the biological process (BP), cellular component (CC) and molecular function (MF) GO-categories are given for each metabolite in Table 2. GO-terms related to the extracellular matrix are enriched towards the top of the lists of transcripts ranked according to correlation to *myo*-inositol and taurine. GO-terms related to the cell cycle, such as "cell cycle process" and "chromosome segregation" are enriched towards the top of the list of transcripts ranked according to correlation to choline. The lists of transcripts ranked according to correlation to glucose, creatine and glycine only gave one enriched GO-term each, "immune system process", "mannosyltransferase activity" and "respiratory chain", respectively. There were no significantly enriched GO-terms for the lists of transcripts ranked according to correlation to GPC and PCho.

**Table 2:** GO enrichment analysis of genes that correlated to the quantified metabolites, using GOrilla.

Metabolite/GO-category	GO-Term	Description	P-value	Enrichment	(N,B,n,b)
**myo-Inositol**					
BP	GO:0032502	developmental process	6.40E-10	1.45	(7307,1397,864,240)
BP	GO:0030198	extracellular matrix organization	1.00E-09	7.16	(7307,56,310,17)
BP	GO:0007155	cell adhesion	1.20E-08	2.45	(7307,377,412,52)
BP	GO:0022610	biological adhesion	1.40E-08	2.44	(7307,378,412,52)
BP	GO:0043062	extracellular structure organization	2.20E-07	4.92	(7307,86,311,18)
MF	GO:0005201	extracellular matrix structural constituent	2.10E-09	7.39	(7307,51,310,16)
CC	GO:0005576	extracellular region	3.80E-21	2.76	(7307,885,311,104)
CC	GO:0044421	extracellular region part	7.10E-17	2.28	(7307,513,757,121)
CC	GO:0031012	extracellular matrix	5.30E-15	5.34	(7307,159,310,36)
CC	GO:0005578	proteinaceous extracellular matrix	2.90E-14	5.57	(7307,142,305,33)
CC	GO:0044420	extracellular matrix part	2.30E-10	3.44	(7307,71,986,33)
CC	GO:0005581	collagen	1.60E-08	21.83	(7307,26,103,8)
**Taurine**					
BP	GO:0032502	developmental process	8.20E-08	1.48	(7307,1397,630,178)
BP	GO:0048731	system development	1.90E-07	2.23	(7307,314,554,53)
MF	GO:0005201	extracellular matrix structural constituent	6.20E-10	16.25	(7307,51,97,11)
CC	GO:0044421	extracellular region part	8.30E-14	2.44	(7307,513,485,83)
CC	GO:0005576	extracellular region	3.90E-12	2.35	(7307,885,267,76)
CC	GO:0031012	extracellular matrix	2.60E-10	3.43	(7307,159,482,36)
CC	GO:0044420	extracellular matrix part	1.80E-09	12.73	(7307,71,97,12)
CC	GO:0005578	proteinaceous extracellular matrix	2.10E-09	3.52	(7307,142,482,33)
CC	GO:0005581	collagen	5.10E-08	9.48	(7307,26,326,11)
**Choline**					
BP	GO:0090304	nucleic acid metabolic process	2.00E-09	1.75	(7307,497,990,118)
BP	GO:0022402	cell cycle process	2.30E-08	2.09	(7307,239,952,65)
BP	GO:0007059	chromosome segregation	3.90E-07	4.65	(7307,25,943,15)
BP	GO:0006139	nucleobase, nucleoside, nucleotide and nucleic acid metabolic process	8.80E-07	1.54	(7307,640,994,134)
CC	GO:0044428	nuclear part	9.80E-11	1.7	(7307,632,999,147)
CC	GO:0005634	nucleus	5.80E-09	1.3	(7307,2062,990,364)
CC	GO:0000775	chromosome, centromeric region	2.50E-08	10.2	(7307,26,303,11)
**Glucose**					
BP	GO:0002376	immune system process	1.60E-09	1.83	(7307,420,999,105)
**Creatine**					
MF	GO:0000030	mannosyltransferase activity	5.80E-08	73.81	(7307,4,99,4)
**Glycine**					
CC	GO:0070469	respiratory chain	5.80E-07	4.74	(7307,22,981,14)

GO-terms in the Biological Process (BP), Molecular Function (MF) and Cellular Compartment (CC) categories that were significantly enriched (p-value < 10E-7) towards the top of the lists of transcripts ranked according to correlation to each of the quantified metabolites. Enrichment is defined as (b/n)/(B/N), where N is the total number of genes, B is the total number of genes associated with a specific GO term, n is the number of genes in the "target set", b is the number of genes in the "target set" associated with a specific GO term [21]. The p-value threshold corresponds to fdr = 8 metabolites * (877 CC GO-terms*10^-7 + 2416 MF GO-terms*10^-7 + 6307 BP GO-terms*10^-7)/31 significant GO terms = 0.003. There were no significantly enriched GO-terms associated with GPC or PCho

### Effect of HR MAS MRS on RNA quality and gene expression

The effect of the HR MAS MRS procedure on RNA integrity and transcription in the breast cancer tissue was assessed separately on 18 pairs of samples. There was not significant evidence that total RNA integrity, quantified by the RIN-value (measured with Bioanalyzer 2100), was affected by HR MAS MRS (p-value = 0.86). The results from the microarray analysis are illustrated in Additional file 3: Plots illustrating the effect of HR MAS MRS on the transcriptome. Hierarchical clustering of the microarray data show that the samples cluster in pairs. Using a false discovery rate of 0.01, 1199 transcripts were significantly differentially expressed in samples that had been subjected to the HR MAS MRS procedure (865 lower and 334 higher, detailed results in Additional file 4: Significantly lower expressed transcripts after HR MAS MRS and Additional file 5: Significantly higher expressed transcripts after HR MAS MRS ). Only 15 of the 1199 transcripts have an estimated log2 fold change larger than 1. Among the 1199 transcripts, several GO-terms were enriched, like "RNA metabolic process" and "Regulation of gene expression" for the transcripts higher expressed after HR MAS MRS and "Protein localization", "Vesicle mediated transport" and "Tricarboxylic acid cycle" for the transcripts lower expressed after HR MAS MRS. The 1199 significantly differentially expressed transcripts were removed from the microarray data of the 46 samples in the main study.

## Discussion

In this study, we have shown the feasibility of merging transcriptomics and metabolomics data from the very same tumor tissue sample. Two strategies of combining microarray data and HR MAS MR spectra are presented, providing a framework for how information from these different molecular levels can be combined and analyzed. We also identified a set of transcripts which showed slightly altered expression after the HR MAS MRS procedure, but overall this variation was smaller than the biological variation in tumors from one patient to another.

In the first strategy to combine gene expression microarray data and HR MAS MR spectra, the expression of "intrinsic" genes was used to classify the samples into established molecular subtypes. The HR MAS MR spectra of the majority of tumor samples, which were classified as luminal A, were further explored to investigate whether metabolic characteristics could define subgroups within a transcriptionally homogenous set of samples. The use of spin echo acquired spectral profiles ensured a more extensive use of metabolic information than using calculated tissue metabolite concentrations, which is limited by several peak areas being non-quantifiable. Three subgroups of luminal A tumors were identified (Figure 2 and 3). The fact that samples cluster together differently with respect to the transcriptional and metabolic profiles (results not shown), indicates that microarrays and HR MAS MRS reflect different traits of the tumors. Lower levels of glucose, which may reflect high energy consumption, and higher levels of alanine in A2 compared with the other luminal A samples indicate that the A2 subgroup has a higher Warburg effect [23]. The lactate signal in A2 also appears to be higher than in the other groups, although not at the significance threshold level. From the GO enrichment analysis, the A2 group was found to be enriched for processes related to cell cycle and DNA repair, compared to A1. The presented subclassification of luminal A might have identified a subgroup of patients (A2) with a more aggressive breast cancer, based on the metabolic and transcriptional profile. However, since this group is small and no long term clinical follow-up is yet available for these patients, a larger cohort with clinical data needs to be analyzed in order to validate whether this finding has clinical impact. It should be noted that intrinsic molecular subtyping is sensitive for selection bias in the cohort analyzed, because of the required gene centring of the microarray data prior to classification. All samples were therefore included in the intrinsic molecular classification, resulting in 10% ER negative samples which is slightly lower than the typical ER negative frequency in IDC. All samples classified as luminal A were ER positive and the majority were PgR positive (3 samples were PgR negative and 1 sample had no IHC data for PgR), which supports the classification since luminal A samples are mostly ER/PgR positive and typically 40-50% of IDCs are classified as luminal A [5]. Even though these preliminary results revealing metabolic subgroups within luminal A tumors need to be reproduced in a larger cohort, they suggest that microarray and HR MAS MRS data complement each other, which can be exploited both in subclassification and for constructing predictors of outcome or treatment response.

The second strategy to combine gene expression microarrays and HR MAS MRS was performed by correlating metabolite concentration and gene expression. In this approach, we have not focused on any specific pathways, but correlated the metabolite concentrations to all transcripts on the microarray that showed some variation across samples. We excluded samples with ER negative status from this analysis to avoid detecting associations related to ER-status, which is known to have a profound effect on the transcriptional profile [24]. The three ER positive samples that were not classified as luminal A were classified as normal-like. Since the gene expression of these three samples also correlated to the published luminal A centroid [17], they were included in the correlation analyses to increase power. The gene transcripts that correlated the most to the concentration of taurine and *myo*-inositol were enriched for GO-terms associated to extracellular processes, which could reflect a tumor-stroma interaction. "Cell adhesion" was also one of the enriched GO-terms for the gene transcripts that correlated to *myo*-inositol, which supports this hypothesis. Taurine and *myo*-inositol are known to be involved in osmoregulation and volume regulation [25]. It should be noted that the concentrations of taurine and *myo*-inositol correlated negatively to tumor percentage (Additional file 2: Scatterplot of metabolite concentrations and tumor percentage), which could contribute to the apparent association of these metabolites to extracellular processes. Gene transcripts that correlated the most to choline concentration were enriched for cell cycle related GO-terms which indicate that the choline level in these samples reflects proliferation. Choline is involved in glycerophospholipid metabolism and is a nutrient taken up by the cells as well as a breakdown product from phosphatidylcholine. The total choline signal can be detected by *in vivo *MRS, and is elevated in breast cancer compared to normal mammary tissue and benign lesions [26]. No significantly enriched GO-terms were found in the genes that correlated the most to the two other choline metabolites involved in the total choline peak, PCho and GPC. Glucose correlated to genes that were significantly enriched for the GO-term "immune system process". Glucose concentration has been shown to have an inverse relationship to the number of proliferating cells [27] and tumor cell density [28]. For creatine, only the GO-term "mannosyltransferase activity", which is a glycosylation process, was significantly enriched among the genes that correlated to the metabolite. The genes that correlated to the amino acid glycine, were also only significantly enriched for one GO-term, "respiratory chain", suggesting a possible association between aerobic respiration and glycine levels in these tumor samples. However, glycine was the only metabolite that showed significant positive correlation to tumor percentage (Additional file 2: Scatterplot of metabolite concentrations and tumor percentage.), which could have influenced this relationship. It is worth noting that few transcripts showed a strong correlation to the eight metabolites, as can be seen in the examples in Figure 5. Since metabolite concentrations reflect the sum of many different pathways, correlating the expression of single genes to metabolites is probably not the optimal way to compare the transcriptional and metabolic profiles of tumors. The fact that the most correlated genes were not directly associated with the metabolic pathways of the metabolites they correlated to also emphasizes the complexity of the relationships between gene expression levels and metabolite concentrations. Improved quantification of tissue metabolite concentrations using ERETIC [27] or dieretic [29] and more refined approaches for data analysis, possibly involving curated metabolic pathways, should be explored in the future when larger datasets of microarray and HR MAS MRS data from the same tumor samples can be obtained, with corresponding clinical information.

MRS and microarray experiments have not previously been performed on the same breast cancer sample from the same patient. A study by Tzika *et al*. combined gene expression microarrays and HR MAS MRS on the same sample of brain tumor and control biopsies [30]. However, no results were reported of combining these two types of information, except for stating that a number of transcripts correlated well to the measured metabolites.

Breast cancer biology is highly complex, which is reflected at many different molecular levels. Using gene expression microarray and HR MAS MRS data from the very same tumor sample can reduce the biological variance which gives a higher power to study the transcriptional and metabolic levels in a combined approach. Even though HR MAS MRS leaves the tumor tissue intact, the procedure exposes the tissue to several potential stresses, including hypoxic conditions and lack of nutrients by being embedded in a surrounding buffer at 4°C for approximately an hour, as well as high centrifugal force and magnetic field during the HR MAS MRS acquisition. In our parallel study to address this issue, total RNA integrity was not significantly affected by HR MAS MRS (p-value = 0.86), and findings in a similar evaluation in prostate tissue support this result [31]. The pairs of tumor samples from each patient that had or had not been analyzed by HR MAS MRS cluster together (Additional file 3: Plots illustrating the effect of HR MAS MRS on the transcriptome), indicating that patient to patient variation is larger than the effect of HR MAS MRS. Even though 1199 transcripts were defined as differentially expressed (fdr < 0.01) by HR MAS MRS, these transcripts showed a small fold change. In their study of prostate tissue, Santos *et al*. reported no differential expression caused by HR MAS MRS [31]. However, Santos *et al*. used cDNA microarray data from unpaired samples to test for differential expression. The patient heterogeneity might have been too high to achieve the power needed to detect possible changes in gene expression in their study.

## Conclusions

Performing HR MAS MRS and microarray analysis on the same sample is feasible, and the effect of HR MAS MRS on the transcriptome was shown to be subtle. Three subgroups of samples within the most prevalent intrinsic subgroup of breast cancer, luminal A, were found using multivariate analyses of HR MAS MRS spectra. One of the subgroups of luminal A samples, designated A2, had metabolic and transcriptional features indicating a higher Warburg effect and more proliferation than the other luminal A groups. Using a different strategy, enrichment analysis of genes with expression levels that correlated to metabolite concentrations revealed different enriched GO-terms associated with specific metabolites. GO-terms related to the extracellular matrix were enriched among the genes that correlated the most to *myo*-inositol and taurine, while cell cycle related GO-terms were enriched among the genes that correlated the most to choline. We have shown that combining transcriptional and metabolic data from the same breast carcinoma sample can contribute to a more refined subclassification of breast cancers as well as reveal relationships between these molecular levels. This study has paved the way for further studies in larger patient cohorts of all subtypes, correlating metabolic subgroups to histopathological characteristics, treatment response and clinical outcome.

## List of Abbreviations

Abbreviations used: CHO: choline; GPC: glycerophosphocholine; PCHO: phosphocholine; IDC: invasive ductal carcinoma; HR MAS MRS: high resolution magic angle spinning magnetic resonance spectroscopy; PBS: phosphate buffered saline; IQR: inter quartile range; PCA: principal component analysis; TSP: trimethylsilyl tetradeuteropropionic acid; FDR: false discovery rate; GO: Gene Ontolgy; MDS: multidimensional scaling; GSA: Gene Set Analysis

## Competing interests

The authors declare that they have no competing interests.

## Authors' contributions

EB carried out HR MAS MRS and microarray experiments and performed microarray preprocessing, data analysis and interpretation of HR MAS MRS and microarray data, and drafted the manuscript. BS performed HR MAS MRS and microarray experiments, did the preprocessing of the HR MAS MRS data, contributed to analysis and interpretation of HR MAS MRS and microarray data and drafting of the manuscript. OCL contributed to analysis and interpretation of HR MAS MRS and microarray data. HJ carried out microarray experiments. TB contributed to interpretation of HR MAS MRS and microarray data. TS contributed to analysis and interpretation of microarray data. SL provided tumor material and clinical data. ALBD conceived of the study, and participated in its design and helped drafting the manuscript. ISG conceived of the study and participated in its design and coordination. All authors revised the manuscript and approved the final version.

## Pre-publication history

The pre-publication history for this paper can be accessed here:

http://www.biomedcentral.com/1471-2407/10/628/prepub

## Supplementary Material

Additional file 1 **Supplementary documentation of exploring the effect of HR MAS MRS on RNA integrity and transcription**. Additional documentation of the experimental procedure and data analysis involving the 18 pairs of samples used in the parallel study to assess the feasibility of performing HR MAS MRS and gene expression microarrays on the same samples. Click here for file

Additional file 2 **Scatterplot of metabolite concentrations and tumor percentage**. The log2 of the tissue concentrations (μmol/gram) of glycerophosphocholine (GPC), glycine, phosphocholine (PCho), creatine, choline, taurine, *myo*-inositol and glucose as well as the tumor percentage (Tumor) are plotted against each other in the upper diagonal panel. Spearman's rank correlation coefficients are given in the lower diagonal panel. Significant (p < 0.05) correlations are indicated by bold font. The values on the axes represent log2 tissue concentrations (μmol/gram), except for the Tumor axis which represents percentage. Click here for file

Additional file 3 **Plots illustrating the effect of HR MAS MRS on the transcriptome**. (A) Hierarchical clustering of the 18 pairs of tumors before (control) and after HR MAS (HRMAS). (B) A plot of the number of significantly differentially expressed (DE) genes caused by HR MAS MRS as a function of false discovery rate. Bonferroni corrected p-value = 0.05 is indicated in the plot. (C) A volcanoplot of significance versus the estimated fold change caused by HR MAS MRS. Transcripts with fdr < 0.01 are colored red or blue to indicate higher or lower expression after HR MAS MRS, respectively. The top Biological Process GO-terms of the differentially expressed genes are listed on each side with the same color-code. Click here for file

Additional file 4 **Significantly lower expressed transcripts after HR MAS MRS**. A summary of the 865 transcripts with significantly lower expression after HR MAS MRS, identified using Limma (R/Bioconductor). The columns ProbeUID, AgilentProbeID, GeneSymbol, SystematicName are annotations as given by Agilent. The columns logFoldChange, AverageExpression, t.statistic, p.value and adjusted.p.value are the results from the modified paired t-tests performed by Limma. Click here for file

Additional file 5 **Significantly higher expressed transcripts after HR MAS MRS**. A summary of the 334 transcripts with significantly higher expression after HR MAS MRS, identified using Limma (R/Bioconductor). The columns ProbeUID, AgilentProbeID, GeneSymbol, SystematicName are annotations as given by Agilent. The columns logFoldChange, AverageExpression, t.statistic, p.value and adjusted.p.value are the results from the modified paired t-tests performed by Limma. Click here for file
