# Supplementary material for: Merging transcriptomics and metabolomics - advances in breast cancer profiling
Source: BMC Cancer. 2010 Nov 16;10:628. doi: 10.1186/1471-2407-10-628 (PMC2996395; doi:10.1186/1471-2407-10-628)
Supplement: Additional file 1 — Supplementary documentation of exploring the effect of HR MAS MRS on RNA integrity and transcription. Additional documentation of the experimental procedure and data analysis involving the 18 pairs of samples used in the parallel study to assess the feasibility of performing HR MAS MRS and gene expression microarrays on the same samples. [file 1471-2407-10-628-S1.PDF]

Supplementary documentation of exploring the effect of HR MAS MRS on RNA integrity and transcription

Sample preparation and experimental design

An additional study was designed to study the effect of HR MAS MRS on RNA quality and gene expression. Fresh frozen tumor tissue from 18 breast cancer patients was split in two similarly sized samples. The first part of the sample was snap frozen in liquid nitrogen and used directly for RNA extraction, while the second part was analyzed by HR MAS MRS and then snap frozen until RNA extraction.

RNA extraction and microarrays

Total RNA was extracted from the fresh frozen tissue following the protocol of the TRIzol reagent (Invitrogen, USA). Total RNA integrity was measured using BioAnalyzer 2100 (Agilent Technologies, USA). The pair of samples from each patient was further processed when the RNA quality of one sample exceeded a RIN-value of 6, suggested by Strand et al. [32]. Total RNA (400 ng) from 18 pairs of samples were used in one-color microarray experiments using 4x44k Agilent Human Whole Genome Oligo Microarrays. The microarrays were scanned using a G2565A scanner and the images were extracted using Agilent Feature Extraction software (v 9.3).

Preprocessing and normalizing the microarray data

The Feature Extraction text files were used directly and analyzed using R (v 2.8) and Bioconductor. The gMeanSignal was used and corrected for multiplicative detrending effects by dividing by the gMultDetrendSignal. The arrays were quantile normalized and log2

transformed using the Limma package (R/Bioconductor) [15]. Control probes, probes which were flagged as outliers on more than 20 % of the arrays, and probes which were flagged as present on less than 50 % of the arrays were removed. The average of duplicate probes was taken, and the probe with the highest IQR was picked when a gene was represented by different probes, leaving 27114 probes.

Testing the effect of HR MAS MRS on RNA integrity and transcription

The effect of HR MAS MRS on RNA integrity was tested using a paired t-test on the RIN-values (measured using Bioanalyzer 2100). Unsupervised hierarchical clustering of the microarray data from the 18 pairs of samples was performed using Eucledian distance and complete linkage. Tests for the effect of HR MAS on gene expression, were performed using Limma (R/ Bioconductor) [15]. The genes that had significantly higher or lower (fdr<0.01) expression in the samples analyzed by HR MAS MRS, were tested for enriched gene ontology (GO) terms [18], using the GOstats package (R/ Bioconductor) [33]. Each of the two genelists were compared to all genes in the filtered microarray data with assigned GO-terms, by using conditional hypergemometric tests to find GO terms that were represented more frequently than expected by chance.

The genes that showed significantly lower expression after HR MAS MRS were enriched for GO-terms related to metabolism, such as “acetyl-CoA metabolic process”, “tricarboxylic acid cycle” and “generation of precursor of precursor metabolites and energy” (see Additional file 3: Plots illustrating the effect of HR MAS MRS on the transcriptome), which suggest a shutdown of metabolism. It is not known if this happens before or during the actual HR MAS MRS acquisition. However, the genes with known function that were higher expressed in samples exposed to HR MAS MRS, were enriched for biological processes such as RNA

splicing and regulation of gene expression. This may indicate that lower expression of several transcripts in the samples exposed to HR MAS MRS is caused by regulation of gene expression in the cells of the tumor tissue, as opposed to random degradation of RNA transcripts.

One limitation to this study is that several samples with or without the HR MAS MRS procedure showed RNA quality below a recommended threshold of 6 [32]. This was done to avoid bias in the analysis of the effect of HR MAS MRS on gene expression, which could arise when excluding samples with poor RNA quality from before or after HR MAS MRS. The low RNA quality in some samples may have introduced additional noise to the data. The false discovery rate calculations are based on the assumption that there is no difference in expression between the samples, which can give an underestimation of the false discovery rate [34]. However, it should be noted that the same test for differential expression was applied to the paired samples with scrambled labels for which sample had been exposed to HR MAS MRS. This test resulted in zero genes passing the fdr<0.05 threshold (results not shown), which supports that the false discovery rate calculations do not simply reflect the heterogeneity of neighboring tissue from the same tumor.
