# Supplementary figures and images for: Merging transcriptomics and metabolomics - advances in breast cancer profiling
Source: BMC Cancer. 2010 Nov 16;10:628. doi: 10.1186/1471-2407-10-628 (PMC2996395; doi:10.1186/1471-2407-10-628)

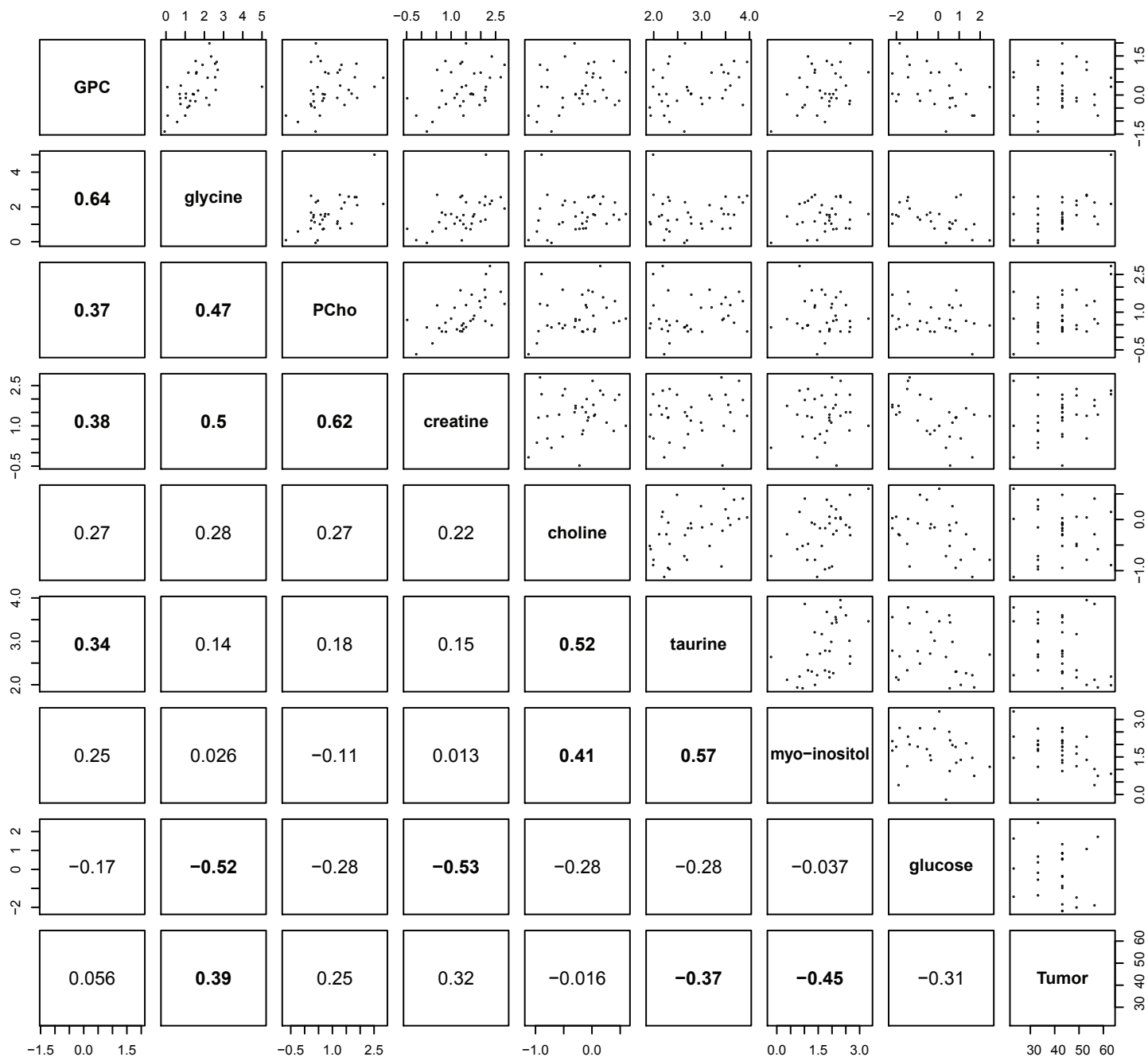

Supplement: Additional file 2 — Scatterplot of metabolite concentrations and tumor percentage. The log2 of the tissue concentrations (μmol/gram) of glycerophosphocholine (GPC), glycine, phosphocholine (PCho), creatine, choline, taurine, myo-inositol and glucose as well as the tumor percentage (Tumor) are plotted against each other in the upper diagonal panel. Spearman's rank correlation coefficients are given in the lower diagonal panel. Significant (p < 0.05) correlations are indicated by bold font. The values on the axes represent log2 tissue concentrations (μmol/gram), except for the Tumor axis which represents percentage. [file 1471-2407-10-628-S2.PDF]
