# Supplementary material for: Merging transcriptomics and metabolomics - advances in breast cancer profiling
Source: BMC Cancer. 2010 Nov 16;10:628. doi: 10.1186/1471-2407-10-628 (PMC2996395; doi:10.1186/1471-2407-10-628)
Supplement: Additional file 3 — Plots illustrating the effect of HR MAS MRS on the transcriptome. (A) Hierarchical clustering of the 18 pairs of tumors before (control) and after HR MAS (HRMAS). (B) A plot of the number of significantly differentially expressed (DE) genes caused by HR MAS MRS as a function of false discovery rate. Bonferroni corrected p-value = 0.05 is indicated in the plot. (C) A volcanoplot of significance versus the estimated fold change caused by HR MAS MRS. Transcripts with fdr < 0.01 are colored red or blue to indicate higher or lower expression after HR MAS MRS, respectively. The top Biological Process GO-terms of the differentially expressed genes are listed on each side with the same color-code. [file 1471-2407-10-628-S3.PDF]

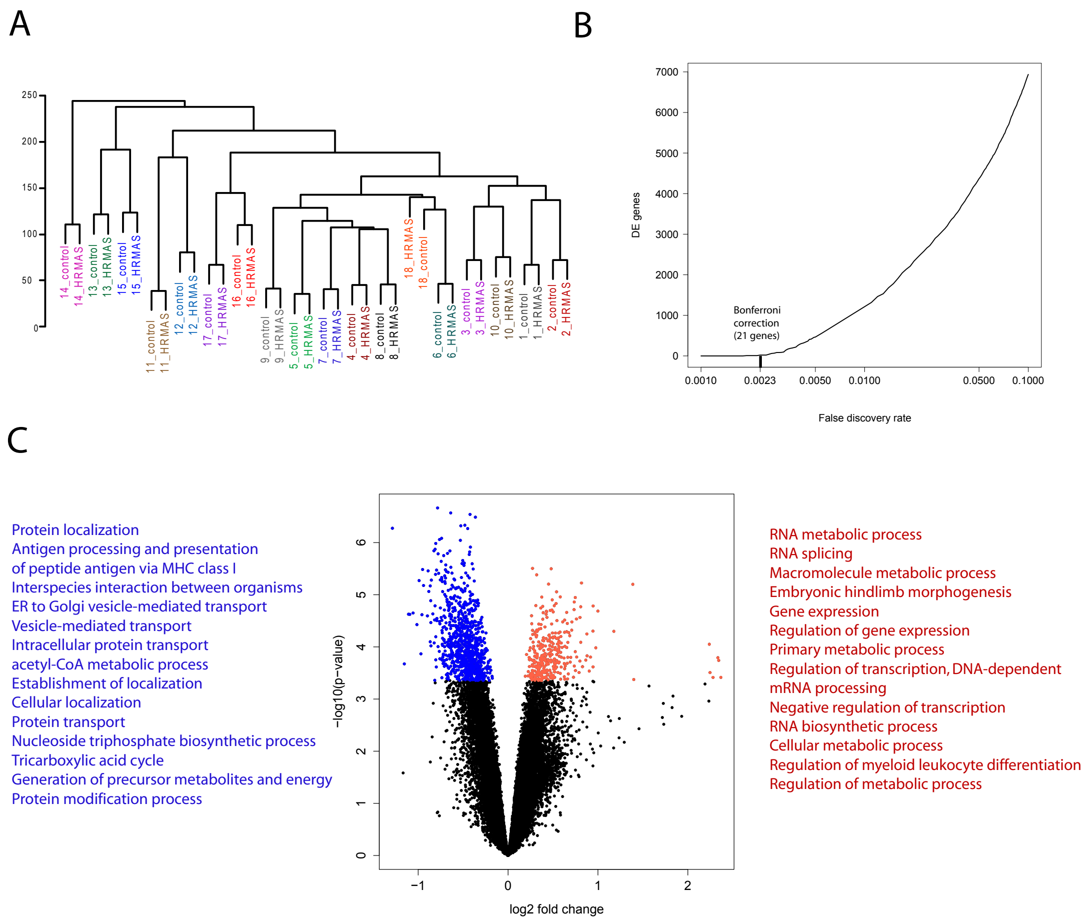

A
250
200
150
100
50
0
14_control
14_HRMAS
13_control
13_HRMAS
15_control
15_HRMAS
11_control
11_HRMAS
12_control
12_HRMAS
17_control
17_HRMAS
16_control
16_HRMAS
9_control
9_HRMAS
5_control
5_HRMAS
7_control
7_HRMAS
4_control
4_HRMAS
8_control
8_HRMAS
18_HRMAS
18_control
6_control
6_HRMAS
3_control
3_HRMAS
10_control
10_HRMAS
1_control
1_HRMAS
2_control
2_HRMAS
B
DE genes
7000
6000
5000
4000
3000
2000
1000
0
Bonferroni correction (21 genes)
0.0010
0.0023
0.0050
0.0100
0.0500
0.1000
False discovery rate
C
Protein localization
Antigen processing and presentation of peptide antigen via MHC class I
Interspecies interaction between organisms
ER to Golgi vesicle-mediated transport
Vesicle-mediated transport
Intracellular protein transport
acetyl-CoA metabolic process
Establishment of localization
Cellular localization
Protein transport
Nucleoside triphosphate biosynthetic process
Tricarboxylic acid cycle
Generation of precursor metabolites and energy
Protein modification process
−log10(p−value)
0
1
2
3
4
5
6
−1
0
1
2
log2 fold change
RNA metabolic process
RNA splicing
Macromolecule metabolic process
Embryonic hindlimb morphogenesis
Gene expression
Regulation of gene expression
Primary metabolic process
Regulation of transcription, DNA-dependent
mRNA processing
Negative regulation of transcription
RNA biosynthetic process
Cellular metabolic process
Regulation of myeloid leukocyte differentiation
Regulation of metabolic process
